# Left Ventricular Diastolic Dysfunction Is Not Associated With Pulmonary Edema in Septic Patients. A Prospective Observational Cohort Study

**DOI:** 10.3389/fcvm.2022.900850

**Published:** 2022-07-01

**Authors:** Ursula Kahl, Leah Schirren, Yuanyuan Yu, Susanne Lezius, Marlene Fischer, Maja Menke, Christoph Sinning, Axel Nierhaus, Maren Vens, Christian Zöllner, Stefan Kluge, Matthias S. Goepfert, Katharina Roeher

**Affiliations:** ^1^Klinik und Poliklinik für Anästhesiologie Universitätsklinikum Hamburg-Eppendorf, Hamburg, Germany; ^2^Institut für Medizinische Biometrie und Epidemiologie Universitätsklinikum Hamburg-Eppendorf, Hamburg, Germany; ^3^Klinik für Intensivmedizin Universitätsklinikum Hamburg-Eppendorf, Hamburg, Germany; ^4^Klinik und Poliklinik für Kardiologie Universitäres Herz- und Gefäßzentrum Universitätsklinikum Hamburg-Eppendorf, Hamburg, Germany; ^5^Institut für Medizinische Biometrie und Statistik Universität zu Lübeck, Universitätsklinikum Schleswig-Holstein Campus Lübeck, Lübeck, Germany; ^6^Klinik für Anästhesie und Intensivmedizin Alexianer St. Hedwigkliniken Berlin, Berlin, Germany

**Keywords:** diastolic dysfunction, lung edema, extravascular lung water, pneumonia, ultrasound, echocardiography, sepsis

## Abstract

**Purpose:**

We aimed to investigate whether left ventricular diastolic dysfunction (LVDD) is associated with pulmonary edema in septic patients.

**Methods:**

We conducted a prospective cohort study in adult septic patients between October 2018 and May 2019. We performed repeated echocardiography and lung ultrasound examinations within the first 7 days after diagnosis of sepsis. We defined LVDD according to the 2016 recommendations of the American Society of Echocardiography and—for sensitivity analysis—according to an algorithm which has been validated in septic patients. We quantified pulmonary edema using the lung ultrasound score (LUSS), counting B-lines in four intercostal spaces.

**Results:**

We included 54 patients. LVDD was present in 51 (42%) of 122 echocardiography examinations. The mean (±SD) LUSS was 11 ± 6. There was no clinically meaningful association of LVDD with LUSS (*B* = 0.55 [95%CI: −1.38; 2.47]; *p* = 0.571). Pneumonia was significantly associated with higher LUSS (*B* = 4.42 [95%CI: 0.38; 8.5]; *p* = 0.033).

**Conclusion:**

The lack of a clinically meaningful association of LVDD with LUSS suggests that LVDD is not a major contributor to pulmonary edema in septic patients.

**Trial Registration:**

NCT03768752, ClinicalTrials.gov, November 30^th^, 2018 - retrospectively registered.

## Introduction

In septic patients, left ventricular diastolic dysfunction (LVDD) is common (1, 2) and associated with weaning from mechanical ventilation (3) and with mortality (1, 2, 4). Septic patients may develop new onset transient LVDD as a sign of sepsis-induced cardiomyopathy (5–7). In septic patients with pre-existing LVDD, LV diastolic function may further aggravate during sepsis.

Endothelial dysfunction with increased vascular permeability is a hallmark of sepsis and can result in pulmonary edema (8). Pulmonary edema is associated with multi-organ dysfunction and mortality (9). LVDD increases hydrostatic pressure and thus potentially aggravates pulmonary edema. In non-septic patients, there is an association between LVDD and hydrostatic pulmonary edema (10–15). Whether there is an association between LVDD and pulmonary edema in septic patients remains uncertain.

We, therefore, aimed to investigate whether septic patients with LVDD—compared to patients with normal LV diastolic function—have more severe pulmonary edema, quantified by the lung ultrasound score (LUSS). Specifically, we tested the hypothesis that LVDD is associated with LUSS in septic patients.

## Patients and Methods

### Study Registration and Ethical Information

We conducted this prospective cohort study between October 2018 and May 2019 in the Department of Intensive Care Medicine (ICU) at the University Medical Center Hamburg-Eppendorf. Ethical approval for this study was provided by the ethics committee of the Hamburg Chamber of Physicians on June 26th, 2018 (reference number PV5769). Patients or their legal representatives provided written informed consent. The study was registered at ClinicalTrials.gov on November 30th, 2018 with the Identifier: NCT03768752. The manuscript adheres to the applicable STROBE guidelines.

### Study Population

Sepsis was defined according to the Sepsis-3 definition (16). Patients were excluded when they were younger than 18 years, had mitral valve disease, persistent or permanent atrial fibrillation, any form of extrinsic cardiac restraint, any implanted mechanical cardiac device, or required extracorporeal membrane oxygenation.

### Ultrasound Examination to Assess LVDD and LUSS

We performed both echocardiography and lung ultrasound daily during the first 7 days after diagnosis of sepsis. Examinations were discontinued earlier, if patients no longer fulfilled sepsis criteria or received palliative care. Ultrasound examinations were conducted by a single experienced investigator (UK). Only images with clearly identifiable anatomic structures and Doppler velocity curves without an angular error above 20° were accepted for interpretation. 2D-images were measured once, in Doppler-images three signals were measured and averaged. Ultrasound images and slopes were analyzed *post hoc* by two independent examiners (UK, LS) and numeric values were averaged. For details on the ultrasound examination see Supplement 1.

The echocardiographic examination of LV diastolic function was in line with the recommendations of the European Society of Intensive Care Medicine (17) and the respective PRICES checklist is available as Supplement 2. We performed echocardiography to assess ejection fraction, stroke volume, Doppler-derived cardiac index, mitral inflow velocity (E- and A-wave), deceleration time of the E-wave, mitral annular tissue velocity (lateral and septal e'- and a'-wave), left atrial maximum volume index, tricuspid regurgitation velocity. We determined and graded LVDD according to the 2016 recommendations of the American Society of Echocardiography (ASE) (18), In patients with preserved LV ejection fraction, LVDD is diagnosed if more than two of the following parameters meet the pathologic threshold: average lateral and septal E/e'-ratio >14, septal e' <7 cm/s or lateral e' <10 cm s^−1^, tricuspid regurgitation velocity >2.8 m s^−1^, and left atrial maximum volume index >34 ml m^−2^. Patients with reduced LV ejection fraction are assumed to have LVDD. In both groups, LVDD is graded in the categories 1, 2, and 3 according to the parameters E/A-ratio (≤ 0.8, >0.8– <2; ≥2), E > 50 cm s^−1^, E/e'-ratio >14, tricuspid regurgitation velocity >2.8 m s^−1^ and left atrial maximum volume index >34 ml m^−2^ (18). For sensitivity analysis, we defined LVDD based on a second algorithm which has been validated specifically for septic patients (19). This algorithm defines LVDD as a septal e' <0.08 m s^−1^, and grades LVDD according to the septal E/e'-ratio in the categories 1 (E/e' ≤ 8), 2 (8> E/e' <13), and 3 (E/e'≥13) (19).

We performed lung ultrasound and used the LUSS to quantify pulmonary edema on a scale of 0–32 by counting and adding B-lines in the intercostal spaces 3/4 and 6/7 on the left and right side during one full breathing cycle (20).

### Statistical Analysis

Statistical calculations were performed with SPSS Version 24 (IBM SPSS Statistics for Windows Released 2016. Armonk, NY: IBM Corp.). All tests were performed on the 5% level. Sensitivity analyses were not adjusted for multiplicity.

Prior to patient enrolment, we calculated group sample sizes of 25 and 25 to detect a difference in LUSS of 5 points with an assumed standard deviation (SD) of 6 in each group at the 0.05 significance level (alpha) with 80% power using a two-sided Mann-Whitney Test. The calculation was performed with the “Inequality Test for Two Means (Simulation)” module of Pass 2008 with 10,000 simulations.

We assessed the association between LVDD and LUSS using a linear mixed model. LUSS was modeled as a metric variable and included as the dependent variable. LVDD was modeled as a binary variable (normal LV diastolic function vs. LVDD grade 1–3) and included as the independent variable of interest. We included clinically relevant potential confounders: age, sex, SOFA score, cardiac index, pneumonia, positive pressure ventilation and fluid balance. Clustering of repeated measurements was accounted for by using a random intercept for the individual patients. The model was gradually reduced following an augmented stepwise backwards approach with respect to changes in parameter estimates ≥10%. Distributional assumptions of the residuals in all linear models were checked with QQ plots. We conducted a sensitivity analysis with LVDD modeled as a categorical variable with four manifestations (normal LV diastolic function, LVDD grade 1, 2, and 3).

Reliability of echocardiography examinations between the two examiners (UK, LS) for the primary endpoint (LUSS), as well for the ultrasound parameters E and septal e' was assessed using intra-class correlation coefficients (ICC).

## Results

**Figure 1:**
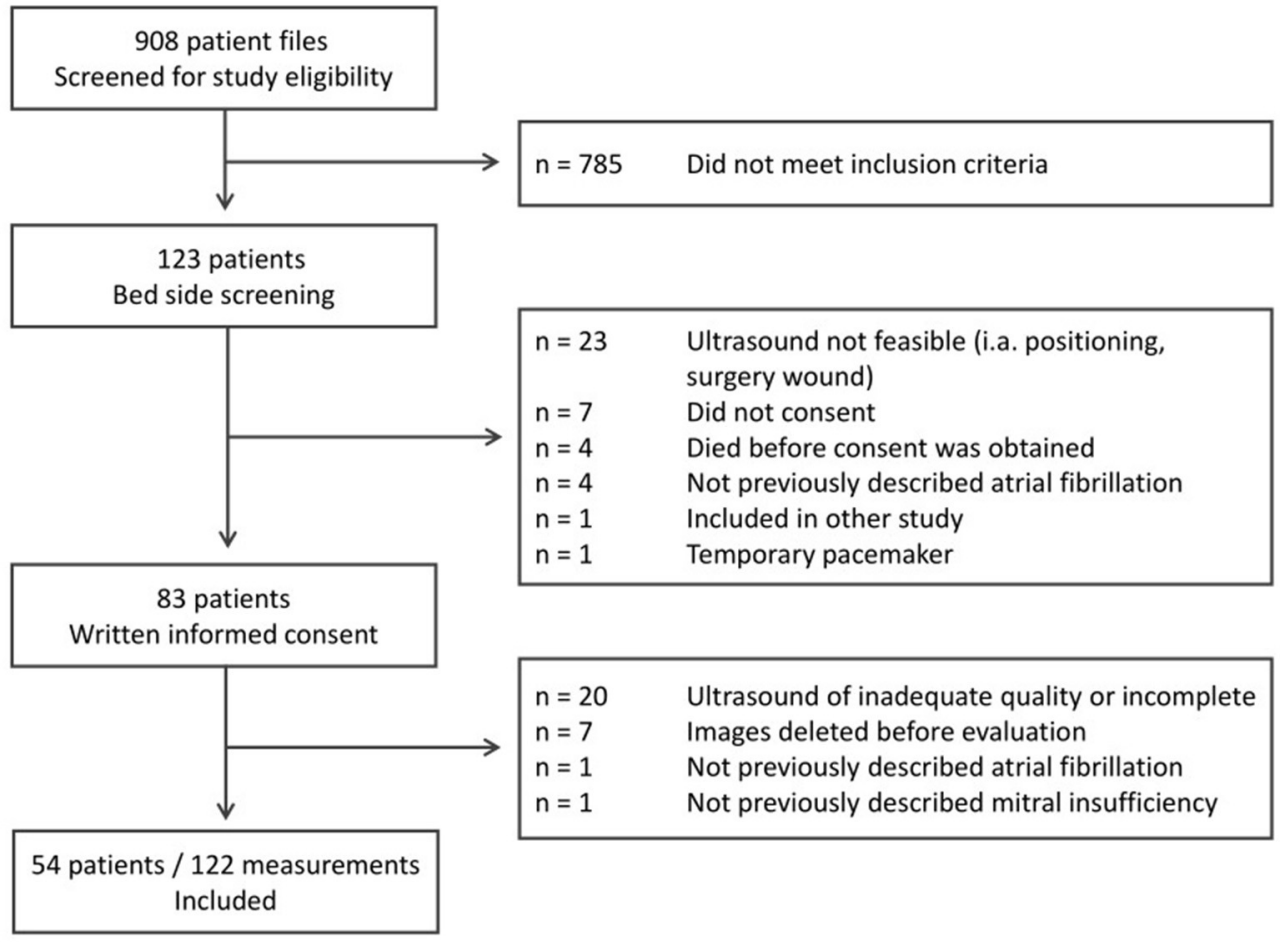
Identification and inclusion of study participants for this prospective observational cohort study.

We analyzed 122 echocardiography examinations in 54 patients (Figure 1 and Tables 1, 2). Details on sepsis severity and therapy are provided in Supplements 2, 3.

**Table 1:** Demographic and medical data.

	**54 patients**	
Sex – female	20	(37)
Age (years)	63	± 16
Body mass index	25.6	± 5.5
Infection site^a^
Lungs	40	(74)
Abdomen	18	(33)
Blood stream	16	(30)
Urinary tract	11	(20)
Bones and soft tissue	5	(9)
Pleura	3	(6)
Mediastinum	3	(6)
Endocardium	3	(6)
Medical history
Oncologic disease	20	(37)
Arterial hypertension	19	(35)
Liver cirrhosis	11	(20)
Chronic liver failure	9	(17)
Chronic kidney disease	9	(17)
Chronic obstructive pulmonary disease	9	(17)
Diabetes mellitus type II	9	(17)
Coronary heart disease	7	(13)
Myocardial infarction	7	(13)
Congestive heart failure	5	(9)
Stroke	5	(9)
Encephalopathy	5	(9)
Peripheral arterial disease	4	(7)
Dementia	4	(7)
Bronchial asthma	4	(7)
Diabetes mellitus type I	1	(2)
ICU mortality	22	(41)

*Data are given in n (%) or mean ± SD*.

*SOFA, Sequential organ failure assessment; COPD, Chronic obstructive pulmonary disease; ICU, Intensive care unit*.

a *Multiple sites possible*.

**Table 2:** Ultrasound examination.

	**122 examinations**
	**(54 patients)**
**Echocardiography**	
LV ejection fraction (%)	49.96 ± 10.89
E wave (m s^−1^)	0.82 ± 0.22
A wave (m s^−1^)	0.82 ± 0.23
E/A	1.06 ± 0.44
e' lateral (m s^−1^)	0.12 ± 0.04
e' septal (m s^−1^)	0.09 ± 0.04
a' lateral (m s^−1^)	0.12 ± 0.04
a' septal (m s^−1^)	0.10 ± 0.04
E/e' lateral	7.39 ± 2.78
E/e' septal	10.12 ± 3.7
E/e' lateral and septal	8.41 ± 2.92
Tricuspid regurgitation vmax (m s^−1^)	2.08 ± 0.64
Left atrial maximum volume index (ml m^−^^2^)	30.62 ± 11.94
Cardiac index^a^ (l min^−1^ m^−2^)	3.42 ± 1.36
**Lung ultrasound**	
Lung ultrasound score ^b^	10.73 ± 6.38

*Data are given in mean ± SD*.

*LV, left ventricular; E, Early mitral flow pattern; A, Atrial mitral flow pattern; E/A, Mitral valve E velocity divided by A-wave velocity; e, early mitral annular tissue velocity lateral or septal, E/e', Mitral valve inflow velocity E divided by mitral annular tissue velocity e'; vmax, maximum velocity*.

a *Doppler-derived*.

b *Enghard et al. (20)*.

**Figure 2:**
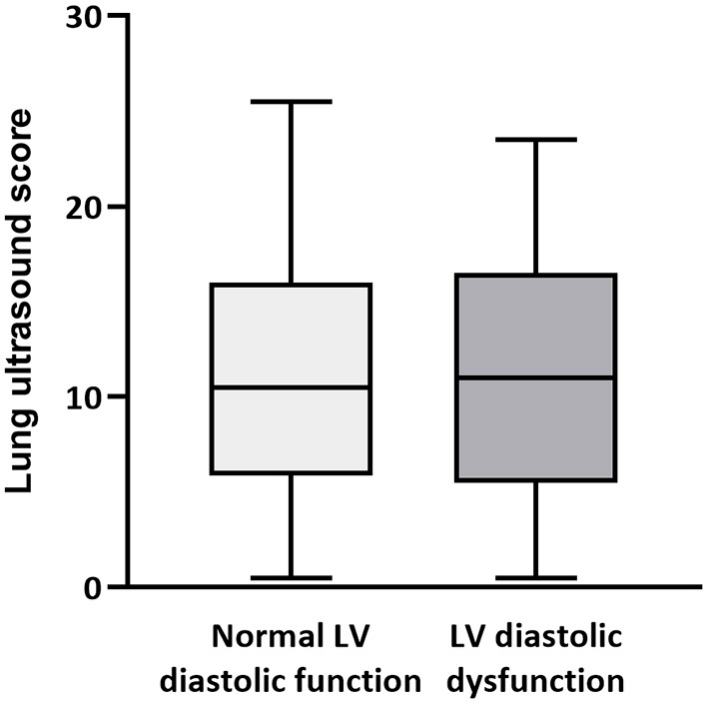
Left ventricular diastolic dysfunction and pulmonary edema. Lung ultrasound score during examinations with normal left ventricular (LV) diastolic function (light gray box) and with LV diastolic dysfunction (dark gray box).

Applying the 2016 ASE recommendations, LVDD was present in 51 (42%) of 122 echocardiography examinations (Supplement 4). The mean (±SD) LUSS was 10.7 ± 6.4; 11.0 ± 6.5 when LVDD was present, and 10.7 ± 6.2 when it was not (Figure 2). There was no clinically meaningful association of LVDD with LUSS (*B* = 0.55 [95%CI: −1.38; 2.47]; *p* = 0.571) (Table 3). Pneumonia was significantly associated with higher LUSS (*B* = 4.42 [95%CI: 0.38; 8.5]; *p* = 0.033; Table 3).

**Table 3:** Linear mixed models.

**Endpoint: lung ultrasound score**	**B**	**CI low**	**CI up**	***p***
**Main analysis**				
**Definition of diastolic dysfunction: ASE Algorithm^a^**				
Normal LV diastolic function vs. LVDD	0.548	−1.375	2.471	0.571
Pneumonia vs. no pneumonia	4.421	0.376	8.467	0.033
No PPV vs. PPV	−1.950	−4.699	0.799	0.162
Age	−0.052	−0.161	0.056	0.340
SOFA score	−0.401	−0.823	0.021	0.062
Cardiac index	−0.320	−1.172	0.532	0.458
**Sensitivity analysis**				
**Definition of diastolic dysfunction: Sepsis-specific Algorithm^b^**				
Normal LV diastolic function vs. LVDD grade 3	2.522	−0.256	5.300	0.075
LVDD grade 1 vs. LVDD grade 3	1.371	−3.278	6.020	0.560
LVDD grade 2 vs. LVDD grade 3	−0.651	−3.664	2.363	0.669
Pneumonia vs. no pneumonia	4.076	0.321	7.831	0.034
Female vs. male sex	2.154	−1.356	5.664	0.224
No PPV vs. PPV	−1.490	−4.133	1.154	0.266
SOFA score	−0.184	−0.573	0.205	0.351
Fluid balance	0.065	−0.223	0.353	0.655

*Linear mixed models: All initial models comprised the variables lung ultrasound score, left ventricular diastolic dysfunction (LVDD), age, sex, sequential organ failure assessment (SOFA) score, cardiac index, pneumonia, positive pressure ventilation (PPV) and fluid balance*.

*B, regression coefficient; CI, confidence interval; E/e' septal, Mitral valve inflow velocity E divided by mitral annular tissue velocity e'*.

a *Nagueh et al. (18);*

b *Lanspa et al. (19)*.

Applying the sepsis-specific LVDD algorithm, LVDD was present in 48 (39%) of 122 echocardiography examinations [LVDD grade 1: 5 (4%); grade 2: 21 (17%) and grade 3: 22 (18%)]. Prevalence of LVDD according to the two different algorithms is displayed in Supplement 4. The sensitivity analysis confirmed the results of the primary analysis (Table 3).

Inter-rater reliability quantified by the average ICC was 0.873 for the LUSS, 0.983 for the E-wave and 0.956 for the septal e'-wave.

## Discussion

We aimed to investigate whether septic patients with LVDD—compared to patients with normal LV diastolic function—have more severe pulmonary edema, quantified by the lung ultrasound score. Contrary to our hypothesis, there was no clinically meaningful association of LVDD with LUSS. Pneumonia was significantly associated with a higher LUSS. These findings were confirmed in the sensitivity analysis using the sepsis-specific definition of LVDD.

Two other studies have investigated the association between LVDD and pulmonary edema in septic patients (21, 22). Both studies defined LVDD based on an elevated E/e'-ratio and used LUSS to quantify pulmonary edema (21, 22). Santos et al. performed one echocardiography and lung ultrasound per patient and—contrary to our results—found an association between LVDD and pulmonary edema (22). The results may differ because only about one-third of their septic patients had a pulmonary source of infection (22). The study by Bataille et al. was restricted to septic patients with acute respiratory distress syndrome due to pneumonia (21). Comparable to our approach, the authors repeatedly performed echocardiography and lung ultrasound (21). In line with our results, there was no association between LVDD and pulmonary edema (21). The association of pneumonia with pulmonary edema presumably masks a clinically meaningful association between LVDD and pulmonary edema (21). Future studies on the association of LVDD with pulmonary edema should thus differentiate between patients with and without pneumonia.

The diagnosis of LVDD in septic patients is challenging. There are no clear diagnostic criteria for LVDD in septic patients (23). Importantly, different echocardiography algorithms may identify different patients as having LVDD (24). The 2016 ASE recommendations (18) are more likely to detect patients with pre-existing LVDD rather than an acute deterioration of diastolic function during sepsis, since they include parameters such as an increased left atrial maximum volume index which expresses a slow-growing adaptation and remodeling of the left atrium due to increased filling pressures (6, 25). To account for the influence of different algorithms, we performed a sensitivity analysis and defined LVDD based on a sepsis-specific algorithm (19). The results confirmed the primary analyses, thus supporting the robustness of our findings.

Our study has limitations. There is no gold standard for the ultrasonographic quantification of pulmonary edema, which limits comparability between studies. LUSS protocols differ regarding the localization and number of examined intercostal spaces (20, 26–28). The LUSS protocol (20) used in this study has several advantages. It has been validated in septic patients (20, 29) and its LUSS values highly correlate with transpulmonary thermodilution-derived extravascular lung water (20), as well as with patient-centered outcomes such as the respiratory distress score or ICU length of stay (30).

According to the 2016 ASE recommendations the vast majority of patients with LVDD in our cohort were classified LVDD grade 1. The physiologic correlate of LVDD grade 1 are elevated filling pressures in the absence of elevated left atrial pressure (6), which may not contribute to pulmonary edema as much as LVDD grade 2 and 3. Future studies should consider comparing patients with normal diastolic function or LVDD grade 1 to patients with LVDD grade 2 or 3. Additionally, future studies should take into account parameters of right ventricular function. Unfortunately, it is not possible to consistently collect information on baseline diastolic function before the onset of sepsis. We thus cannot reliably distinguish between patients with pre-existing and new onset LVDD.

Our patient cohort was heterogeneous in regard to pre-existing conditions, infection sites, microbial spectrum and sepsis therapy. We aimed to control for this heterogeneity by adjusting the analysis for potential confounders. Most importantly, we included the SOFA score in the analysis to account for sepsis severity. Potential confounders which are part of the SOFA Score such as arterial blood pressure, vasopressor support, PaO_2_/FiO_2_ ratio, serum creatinine where not included as individual variables in addition to the SOFA score. Additionally, we adjusted for the cardiac index to account for systolic function and for the daily fluid balance to account for iatrogenic fluid supply or extraction in patients with and without kidney failure and renal replacement therapy. We adjusted for positive pressure ventilation to account for respiratory failure and for pneumonia to account for the pneumonia-associated risk of lung edema. As we only included 54 patients, we could not compare subgroups of patients with and without pneumonia. Future studies should differentiate between patients with and without pneumonia.

A major strength of our study is that ultrasound examinations were standardized and performed by a single examiner and two independent evaluators, showing excellent inter-rater reliability.

## Conclusion

The lack of a clinically meaningful association of LVDD with LUSS suggests that LVDD is not a major contributor to pulmonary edema in septic patients.

## Data Availability Statement

The raw data supporting the conclusions of this article will be made available by the authors, without undue reservation.

## Ethics Statement

The studies involving human participants were reviewed and approved by Ethics Comittee of the Hamburg Chamber of Physicians Ärztekammer Hamburg Weidestr. 122 b 22083 Hamburg. The patients/participants or their legal representatives provided their written informed consent to participate in this study.

## Author Contributions

UK: conception and design of the work, acquisition, analysis and interpretation of data, and writing of the original draft. LS: acquisition and writing of the original draft. YY: acquisition and substantial revision of the manuscript. MM: writing original draft. SL: analysis and substantial revision of the manuscript. MF: analysis and interpretation of data and substantial revision of the manuscript. CS, SK, and AN: substantial revision of the manuscript. MV and CZ: design of the work, substantial revision of the manuscript. MG: conception and design of the work and substantial revision of the manuscript. KR: conception and design of the work, interpretation of data, and writing of the original draft. All authors have approved the submitted version (and any substantially modified version that involves the author's contribution to the study), and have agreed both to be personally accountable for the author's own contributions and to ensure that questions related to the accuracy or integrity of any part of the work, even ones in which the author was not personally involved, are appropriately investigated, resolved, and the resolution documented in the literature.

## Funding

UK was funded by the Clinician Scientist Program of the medical faculty of the University of Hamburg, during the conduct of the study. The University of Hamburg was not involved in any of the following: study design, conduct of the research, preparation of this manuscript, analysis and interpretation of data; writing of the report; decision to submit the article for publication. MF receives financial support from the Johanna und Fritz Buch Gedächtnis-Stiftung. The Johanna und Fritz Buch Gedächtnis-Stiftung was not involved in any of the following: study design, conduct of the research, preparation of this manuscript, analysis and interpretation of data; writing of the report; decision to submit the article for publication.

## Conflict of Interest

The authors declare that the research was conducted in the absence of any commercial or financial relationships that could be construed as a potential conflict of interest.

## Publisher's Note

All claims expressed in this article are solely those of the authors and do not necessarily represent those of their affiliated organizations, or those of the publisher, the editors and the reviewers. Any product that may be evaluated in this article, or claim that may be made by its manufacturer, is not guaranteed or endorsed by the publisher.
