# Supplementary material for: Left Ventricular Diastolic Dysfunction Is Not Associated With Pulmonary Edema in Septic Patients. A Prospective Observational Cohort Study
Source: Front Cardiovasc Med. 2022 Jul 1;9:900850. doi: 10.3389/fcvm.2022.900850 (PMC9283750; doi:10.3389/fcvm.2022.900850)
Supplement: Supplementary file 1 [file Data_Sheet_1.PDF]

Supplement 1. Ultrasound examination method

Parameter	Probe	Mode	View	Calculation
**Echocardiography**				
Ejection Fraction	Sector probe	B-mode	A4C; A2C	Biplane method of discs; modified Simpson's rule
Stroke volume	Sector probe	B-mode; PW Doppler	PLAX; A5C	Left ventricular outflow tract (LVOT) diameter (cm); LVOT subvalvular velocity time integral (cm)
Mitral inflow pattern	Sector probe	PW Doppler	A4C	E-wave; A-wave (m s^{-1}) Deceleration time of the E-wave (ms)
Mitral annular tissue velocity	Sector probe	Tissue Doppler	A4C	Peak lateral and septal velocity during early and the atrial filling phase (lateral and septal e'- and a'-wave; m s^{-1})
Left atrial maximum volume index	Sector probe	B-mode	A4C; A2C	Biplane method of discs; modified Simpson's rule; indexed to Body surface area (ml m^{-2})
Tricuspid regurgitation velocity	Sector probe	CW Doppler	A4C	Maximum velocity (m s^{-1})
**Lung ultrasound**				
Lung ultrasound score[1]	Curved array	B-mode	longitudinal view, ICS 3/4, ICS 6/7 left and right	Number of B-lines during one full breathing cycle. (ICS Score 0-8; total score 0-32)

Supplement 1. A2C/A4C/A5C = Apical 2/4/5-chamber view; PLAX = parasternal long axis; PW = pulsed wave; CW = continuous wave; ICS = intercostal space.

[1] Enghard P, Rademacher S, Nee J, Hasper D, Engert U, Jorres A, et al. Simplified lung ultrasound protocol shows excellent prediction of extravascular lung water in ventilated intensive care patients. Crit Care. 2015 Feb 6;19:36.

Kahl et al.: Diastolic dysfunction and pulmonary edema during sepsis
Supplemental material 2: PRICES Checklist[1]

	Checklist items	LV systolic function	RV function	LV diastolic function	Fluid management
**A1**	**Research vs clinical study**				
	• Research study			X	
	• Clinical study				
**A2**	**Study information**				
	• Specific study type			X	
	• State study design			X	
	• Report sample size			X	
**A3**	**Patient information**				
	• Age			X	
	• Gender			X	
	• Height & weight (or BMI)			X	
	**Comorbidities**				
	• Ischaemic heart disease			X	
	• Atrial fibrillation			X	
	• Hypertension			X	
	• HFpEF				
	• HFrEF				
	• Pacemaker implant present			X	
	• COPD or pulmonary hypertension			X	
	• CKD or hemodialysis			X	
**A4**	**Echocardiography information**				
	• Type of echo (TTE or TEE)			X	
	• Indicate if data collected at end-expiration				
	• No. of beats used for averaging			X	
	• Report vendor of ultrasound machine			X	
	• Indicate if airway pressure trace displayed on screen				
**A5**	**Clinical information at the time of echo**				
**A5.1**	***Ventilation***				
	• Mode of ventilation			X	
	• Tidal volume				
	• Plateau pressure				
	• PEEP			X	
**A5.2**	***Hemodynamics***				
	• Cardiac rhythm & heart rate			X	
	• BP			X	
	• Inotropes, vasopressors and doses			X	
**A6**	**Reliability** (for research study)				
	• Feasibility of echo stated			X	
	• Intra-observer variability				
	• Inter-observer variability			X	
	• Indicate if observer blinded to treatment, if applicable				
**A7**	**Statistics** (for research study only)				
	• Sample size calculation			X	
	• Indicate if statistician blinded to treatment / group				
	• Address confounders, if applicable			X	
	• Internal validation provided, if applicable				

[1]Sanfilippo F, Huang S, Herpain A, Balik M, Chew MS, Clau-Terré F, et al. The PRICES statement: an ESICM expert consensus on methodology for conducting and reporting critical care echocardiography research studies. Intensive Care Med 2020. https://doi.org/10.1007/s00134-020-06262-5.

Kahl et al.: Diastolic dysfunction and pulmonary edema during sepsis
Supplemental material 2: PRICES Checklist[1]

LV systolic function	B1	**LV systolic function indices**	
		• LV ejection fraction	X
		• Tissue Doppler S' velocity	
		• Mitral annular systolic plane excursion (MAPSE)	
		• LV strain or strain rate	
	B2	**LV size**	
		• LV end-diastolic diameter or volume	
	B3	**Other functional indices to aid interpretation**	
		• Cardiac output	X
		• Stroke volume	X
		• Any heart valve dysfunction	

RV systolic function	C1	**RV systolic function indices**	
		• Tricuspid annular systolic plane excursion (TAPSE)	
		• RV fractional area change	
		• Tissue Doppler S' velocity	
		• LV strain or strain rate	
	C2	**RV size and wall thickness**	
		• RV end-diastolic diameter or area	
		• RV:LV end-diastolic area ratio	
		• RV wall thickness	
	C3	**Other functional indices to aid interpretation**	
		• PFO or other shunt(s)	
		• Pericardial effusion	
		• Paradoxical septal motion	
		• Inter-atrial septal bowing	
		• IVC diameter	

LV diastolic function	D1	**Indices for evaluation of LV diastolic function**	
		• E/A ratio	X
		• Tissue Doppler E' velocity	X
		• E/E' ratio	X
		• PAP or TR peak velocity	X
		• LA size	X
		• Mitral E deceleration time	
		• Pulmonary venous flow	
	D2	**Other functional indices to aid interpretation**	
		• BP: systolic, diastolic and mean	X
		• Related chronic medications	
	D3	**Criteria used for grading diastolic function**	
		• State or quote criteria	X
		• Cite reference	X
		• Technical details of measurements	X

Fluid management	E1	**Evaluation of fluid management**	
		• Define the meaning of FR clearly	
		• State parameter(s) used for predicting fluid responsiveness (FR)	
		• Describe parameters used to assess FR (e.g. cut-offs)	
	E2	**Other information to aid interpretation** (research study only)	
		• State reference standard used in diagnostic or validation study	
		• State if echo is used to measure the reference value (e.g. CO)	
		• State technical information on echo measurements	
		• Describe any procedures used for FR assessment	

[1]Sanfilippo F, Huang S, Herpain A, Balik M, Chew MS, Clau-Terré F, et al. The PRICES statement: an ESICM expert consensus on methodology for conducting and reporting critical care echocardiography research studies. Intensive Care Med 2020. https://doi.org/10.1007/s00134-020-06262-5.

Supplement 3. Sepsis severity and disease progression

	54 patients (122 examinations)
**Disease progression**	
Maximum SOFA score	11± 4
Maximum Lactate (mmol l^{-1})	3.3 ± 3.3
Heart rate (bpm)	92 ± 19
MAP (mmHg)	73 ± 13
Temperature (°C)	37 ± 1
Horowitz Index	193 ± 8
Septic shock[a]	54 (44)
**Catecholamines**	
Mean dose of Norepinephrine (µg kg^{-1} min^{-1})[b]	0.325 ± 0.443
Norepinephrine (n)	109 (89)
Dobutamine[c] (n)	8 (7)
Adrenaline[c] (n)	2 (2)
No catecholamines (n)	13 (11)
**Sedation**	
No sedation (n)	56 (46)
iv and inhalational sedation (n)	56 (46)
Inhalational sedation (n)	6 (5)
iv sedation	4 (3)
**Ventilation**	
No positive pressure ventilation (n)	44 (36)
Positive pressure ventilation (n)	78 (64)
PEEP (mbar)[d]	5.6 ± 4.3
**Volume resuscitation**	
Fluid balance (litre/24hours)	2.5 ± 3.0
Renal replacement therapy (n)	36 (30)
Medication with diuretics (n)	28 (23)

Supplement 3: Data are given in n (%) or mean ± SD. [a]Defined as lactate > 2 mmol/L and vasopressor support[1]. [b]Highest in 24 hours. [c]in addiation to norepinephrine. [d]Patients with HFNC received an estimated PEEP of 3 mbar[2]. SOFA score = Sequential organ failure assessment score; HFNC = high flow nasal cannula; MAP = mean arterial pressure; PEEP = positive end-expiratory pressure.

[1] Singer M, Deutschman CS, Seymour CW, Shankar-Hari M, Annane D, Bauer M, et al. The Third International Consensus Definitions for Sepsis and Septic Shock (Sepsis-3). JAMA. 2016 Feb 23;315(8):801.
[2] R. Parke, S. McGuinness, and M. Eccleston, 'Nasal High-Flow Therapy Delivers Low Level Positive Airway Pressure', *British Journal of Anaesthesia* 103, no. 6 (December 2009): 886–90, https://doi.org/10.1093/bja/aep280

Supplement 4. Sepsis therapy

Patients were treated according to national and international guidelines.[1,2] They received anti-infective medication depending on the suspected microbial spectrum and infection site. Arterial blood pressure was measured continuously in all patients with an arterial catheter. In patients with sepsis-induced tissue hypoperfusion and suspected hypovolemia, crystalloid fluids were administered using the fluid challenge technique.[2] Vasoactive medication (norepinephrine, dobutamine, and epinephrine) was administered with a targeted mean arterial blood pressure above 65mmHg. In patients with renal failure and indication for dialysis such as hypervolemia, hyperkalaemia, or refractory metabolic acidosis, continuous renal replacement therapy was initiated. Patients with hypoxemic respiratory failure received either high-flow oxygen through a nasal cannula (HFNC),[3] non-invasive, or invasive ventilation depending on the severity of the respiratory failure.[4] The positive end-expiratory pressure (PEEP) was titrated according to the recommendations of the acute respiratory distress syndrome (ARDS) network.[5] If sedation was necessary, patients received either propofol, midazolam or inhaled isoflurane with a targeted score of 0 or -1 on the Richmond Agitation and Sedation Scale.[6]

---

1. Deutsche Sepsis Gesellschaft e. V., Brunkhorst FM, Weigand MA, Pletz M, Gastmeier P, Lemmen SW, et al. S3-Leitlinie Sepsis – Prävention, Diagnose, Therapie und Nachsorge: Langfassung. Med Klin - Intensivmed Notfallmedizin. 2020 May;115(S2):37–109.

2. Rhodes A, Evans LE, Alhazzani W, Levy MM, Antonelli M, Ferrer R, et al. Surviving Sepsis Campaign: International Guidelines for Management of Sepsis and Septic Shock: 2016. Intensive Care Med. 2017 Mar;43(3):304–77.

3. Parke R, McGuinness S, Eccleston M. Nasal high-flow therapy delivers low level positive airway pressure. Br J Anaesth. 2009 Dec;103(6):886–90.

4. Arbeitsgemeinschaft der Wissenschaftlichen Medizinischen Fachgesellschaften (AWMF), Deutsche Gesellschaft für Anästhesiologie und Intensivmedizin (DGAI). S3-Leitlinie Invasive Beatmung und Einsatz extrakorporaler Verfahren bei akuter respiratorischer Insuffizienz. AWMF Leitlinien-Regist Nr 001021. 2017 Dec 4;

5. The Acute Respiratory Distress Syndrome Network. Ventilation with Lower Tidal Volumes as Compared with Traditional Tidal Volumes for Acute Lung Injury and the Acute Respiratory Distress Syndrome [Internet]. http://dx.doi.org/10.1056/NEJM200005043421801. Massachusetts Medical Society; 2009 [cited 2021 Apr 17]. Available from: https://www.nejm.org/doi/10.1056/NEJM200005043421801

6. Ely EW, Truman B, Shintani A, Thomason JWW, Wheeler AP, Gordon S, et al. Monitoring Sedation Status Over Time in ICU Patients: Reliability and Validity of the Richmond Agitation-Sedation Scale (RASS). JAMA. 2003 Jun 11;289(22):2983.

Supplement 5: Comparison of the prevalence of left ventricular diastolic dysfunction according to different algorithms

	2016 ASE recommendations[3]				
	Normal LV diastolic function	LVDD grade 1	LVDD grade 2	LVDD grade 3	LVDD indeterminate
**Sepsis-specific algorithm[4]**					
Normal LV diastolic function	35 (29)	29 (24)	0 (0)	0 (0)	10 (8)
LVDD grade 1	3 (2)	2 (2)	0 (0)	0 (0)	0 (0)
LVDD grade 2	13 (11)	7 (6)	0 (0)	0 (0)	1 (1)
LVDD grade 3	3 (2)	7 (6)	5 (4)	1 (1)	6 (5)

Supplement 5: Data are given in n (%). ASE = American society of echocardiography; LV = left ventricular; LVDD = Left ventricular diastolic dysfunction.

[3] Nagueh SF, Smiseth OA, Appleton CP, Byrd BF, Dokainish H, Edvardsen T, Flachskampf FA, Gillebert TC, Klein AL, Lancellotti P, Marino P, Oh JK, Alexandru Popescu B, Waggoner AD, Houston, Texas; Oslo, Norway; Phoenix, Arizona; Nashville, Tennessee; Hamilton, Ontario, Canada; Uppsala, Sweden; Ghent and Liège, Belgium; Cleveland, Ohio; Novara, Italy; Rochester, Minnesota; Bucharest, Romania; and St. Louis, Missouri. Recommendations for the Evaluation of Left Ventricular Diastolic Function by Echocardiography: An Update from the American Society of Echocardiography and the European Association of Cardiovascular Imaging. Eur Heart J – Cardiovasc Imaging. 2016 Dec;17(12):1321–60.

[4] Lanspa MJ, Gutsche AR, Wilson EL, Olsen TD, Hirshberg EL, Knox DB, Brown SM, Grissom CK. Application of a simplified definition of diastolic function in severe sepsis and septic shock. Crit Care [Internet]. 2016 Dec [cited 2019 Mar 7];20(1).
Available from: http://ccforum.biomedcentral.com/articles/10.1186/s13054-016-1421-3
